# Loss of β-Glucocerebrosidase Activity Does Not Affect Alpha-Synuclein Levels or Lysosomal Function in Neuronal Cells

**DOI:** 10.1371/journal.pone.0060674

**Published:** 2013-04-08

**Authors:** Georgia Dermentzaki, Evangelia Dimitriou, Maria Xilouri, Helen Michelakakis, Leonidas Stefanis

**Affiliations:** 1 Division of Basic Neurosciences, Biomedical Research Foundation of the Academy of Athens, Athens, Greece; 2 Department of Enzymology and Cellular Function, Institute of Child Health, Athens, Greece; 3 Second Department of Neurology, National and Kapodistrian University of Athens Medical School, Athens, Greece; UCL Institute of Neurology, United Kingdom

## Abstract

To date, a plethora of studies have provided evidence favoring an association between Gaucher disease (GD) and Parkinson’s disease (PD). GD, the most common lysosomal storage disorder, results from the diminished activity of the lysosomal enzyme β-glucocerebrosidase (GCase), caused by mutations in the β-glucocerebrosidase gene (GBA). Alpha-synuclein (ASYN), a presynaptic protein, has been strongly implicated in PD pathogenesis. ASYN may in part be degraded by the lysosomes and may itself aberrantly impact lysosomal function. Therefore, a putative link between deficient GCase and ASYN, involving lysosomal dysfunction, has been proposed to be responsible for the risk for PD conferred by GBA mutations. In this current work, we aimed to investigate the effects of pharmacological inhibition of GCase on ASYN accumulation/aggregation, as well as on lysosomal function, in differentiated SH-SY5Y cells and in primary neuronal cultures. Following profound inhibition of the enzyme activity, we did not find significant alterations in ASYN levels, or any changes in the clearance or formation of its oligomeric species. We further observed no significant impairment of the lysosomal degradation machinery. These findings suggest that additional interaction pathways together with aberrant GCase and ASYN must govern this complex relation between GD and PD.

## Introduction

Gaucher Disease (GD), is the most prevalent lysosomal storage disorder (estimated incidence 1 in 40.000–60.000) that follows an autosomal recessive trait. It is due to the diminished activity of the enzyme β-glucocerebrosidase (GCase). As a consequence glucosylceramide (GluCer) and, its deacylated form, glucosylsphingosine (GlcSph), accumulate in practically every tissue examined, including the brain. This accumulation is believed to lead to cellular pathology, through a mechanism that is not completely understood. The disease exhibits a remarkable heterogeneity that has been observed in its clinical symptoms, even among twins carrying identical mutations, suggesting the involvement of additional modifiers [1], [2]. Classically, GD is divided into three clinical types, GD type 1, GD type 2 and GD type 3 depending on the presence, degree and rate of progression of neurologic involvement. GD 1, the most common type, is distinguished as the non-neuronopathic type, a classification that has been challenged due to its association with Parkinson’s Disease (PD) and parkinsonism [3]. More specifically, genetic studies have identified GBA mutations as one of the most common reported risk factors for PD [4]–[12]. Many studies have attempted to shed light on the complex relation between GD and PD. One candidate culprit for this relation is alpha-synuclein (ASYN). Firstly, ASYN is an abundant neuronal protein that has been linked to PD pathogenesis. Intraneuronal inclusions, mainly consisting of ASYN (Lewy bodies, Lewy neurites), are pathological hallmarks of PD and other synucleinopathies. Secondly, ASYN degradation has been proposed to take place in lysosomes, in part via chaperone-mediated autophagy (CMA) [13]–[16]; therefore, potential lysosomal dysfunction in neuronal cells due to diminished GCase activity could in time lead to ASYN accumulation and PD pathology.

In support to the presence of an interaction (direct or indirect) between ASYN and aberrant GCase, gain and/or loss-of-function models have been proposed. Immunofluorescence studies in brain samples from PD and/or Lewy body dementia individuals, along with GD or GBA mutation carriers, have shown the presence of GCase in LBs and LNs in most cases [17], [18], suggesting a gain-of-function mechanism via a direct interaction. In accordance with the above, a recent study has demonstrated aggregation of ASYN in brain samples from individuals with GBA mutations [19]. Another study revealed that the direct interaction between WT GCase and ASYN under acidic conditions was stronger compared to the interaction with mutant GCase [20]. Likewise, GBA mutants were reported to promote ASYN accumulation whereas pharmacological inhibition of GCase activity did not affect ASYN levels *in vitro* [21]. On the other hand, other groups favor a positive correlation between loss of GCase activity and ASYN accumulation. Manning Bog et al [22] showed elevated ASYN levels following pharmacological inhibition of GCase. In support of the notion of loss of function, in an extended study, Mazzulli et al [23] demonstrated accumulation of toxic, soluble oligomeric intermediates of ASYN and impaired lysosomal function, both in primary neuronal cultures, in which GBA had been lowered via RNAi, and in human iPS neurons derived from a GBA mutation carrier. Under the scope of this evidence, we undertook the present study to further investigate the contribution of deficient GCase activity to ASYN levels and lysosomal function in differentiated SH-SY5Y cells, in a doxycycline-inducible Tet-Off system for ASYN, and in primary neuronal cultures. We found that GCase deficiency was not sufficient to either up-regulate ASYN or compromise the lysosomal degradation machinery, suggesting the presence of additional molecular mechanisms which, synergistically with aberrant GCase activity, contribute to the pathogenesis of PD and related synucleinopathies.

## Materials and Methods

### Cell Culture

Neuroblastoma SH-SY5Y cells were cultured in RPMI 1640 plus L-glutamine (Sigma, St. Louis, MO, USA) supplemented with 10% heat-inactivated FBS and 1% penicillin/streptomycin (complete medium). The stable inducible Tet-Off SH-SY5Y neuroblastoma cell line overexpressing WT ASYN described in [14], [24] was also cultured in the above complete medium in the presence (WT+dox) or in the absence (WT – dox) of doxycycline (2 µg/mL) (dox, Clontech). Differentiation of SH-SY5Y cells was with all-trans Retinoic Acid (RA, 20 mM, Sigma). With the particular SH-SY5Y line we are working with, there is full neuronal differentiation, as assessed by neuronal markers, 5 days after RA application [24]. For pharmacological studies Conduritol B- Epoxide (CBE, Sigma), 3-Methyladenine (3MA, Sigma-Aldrich), Bafilomycin (Baf, Sigma-Aldrich), NH_4_Cl (Sigma-Aldrich) and rapamycin (rap, Sigma-Aldrich) were added at indicated time points and concentrations. In particular, the CBE inhibitor was replenished every other day together with the change of the medium.

### Primary Culture

Cultures of Wistar rat (embryonic day 18) cortical neurons were prepared as previously described [25], [26]. 1.5×10^6^ cells were plated onto poly-D-lysine-coated six-well dishes. Cells were maintained in Neurobasal medium (Gibco, Rockville, MD, USA; Invitrogen, Carlsbad, CA, USA), with B27 serum-free supplement (Gibco; Invitrogen), L-glutamine (0,5 mM), and penicillin/streptomycin (1%). More than 98% of the cells cultured under these conditions represent post-mitotic neurons [27]. All efforts were made to minimize animal suffering and to reduce the number of the animals used, according to the European Communities Council Directive (86/609/EEC) guidelines for the care and use of laboratory animals. All animal experiments were approved by the Institutional Animal Care and Use Committee of Biomedical Research Foundation of the Academy of Athens.

### Assay of GCase Activity

Cell pellets from differentiated SH-SY5Y cells or rat embryonic cortical cultures treated with or without CBE were homogenized in water by sonication. GCase activity was determined in whole cell homogenates using the artificial fluorogenic substrate 4-methylumbelliferyl β-D-glucopyranoside in the absence of taurocholate. The reaction mixture contained 5 mmol/L substrate in citrate phosphate buffer pH:4,5. The reaction was stopped after 30 min incubation at 37°C by the addition of 0,25 mol/L glycine-NaOH, pH:10,4 [28].

### Fractionation Assay

Stable inducible Tet-Off SH-SY5Y neuroblastoma cell lines overexpressing WT ASYN [14], [29] were cultured in the presence (+) or absence (−) of dox (2 µg/mL) for 7 days. Subsequently, cells were differentiated for 5 days and then were exposed for various periods of time to CBE (200 µM), a pharmacological GCase inhibitor, either in the presence or absence of dox. Untreated cells were also used (ctl). Following this, cells were lysed in mild lysis buffer (50 mM Tris, pH 7,6, 1 mM EDTA, PH 8,0) and protease inhibitors. Lysates were passed through an insulin syringe and centrifuged at 600×g for 5 min at 4°C. Supernatant was transferred to a new tube and centrifuged at 100.000×g for 2 h at 4°C. The resultant supernatant represents the cytosolic fraction. The pellet was washed ×5 with solution: 50 mM Tris, pH 7,6, 1 M NaCl, 1 mM EDTA, PH 8,0 and centrifuged at 10.000 X g for 5 min at 4°C. Finally the pellet was resuspended in STET lysis buffer (50 mM Tris, pH 7,6, 150 mM NaCl, 1% Triton X-100, 2 mM EDTA) with protease inhibitors. This fraction represents the membrane-associated, Triton X-100 soluble fraction [30].

### Intracellular Protein Degradation

Total protein degradation in differentiated SH-SY5Y cultured cells was measured by pulse-chase experiments [13], [14], [29], [31] with modifications. Briefly, differentiated SH-SY5Y cells were exposed to CBE (200 µM) at day 5 of differentiation for 5 days. Untreated cells were used as well as controls. Following this treatment, cells were labeled with [^3^H] leucine (2 µCi/ml) (leucine, L-3,4,5; PerkinElmer Life Sciences) at 37°C for 48 h, in the presence or absence of CBE. The cultures were then extensively washed with medium and returned in starvation medium (0,5% FBS) containing 2,8 mM of unlabeled excess leucine for 6 h. This medium containing mainly short lived proteins was removed and replaced with complete fresh medium containing the general lysosomal inhibitor Baf (500 nM)/NH_4_Cl (20 mM) or the inhibitor of macroautophagy 3MA (10 mM) in the presence or absence of CBE (200 µM), as above. At this concentration 3MA is known to completely inhibit macroautophagy [32], [33] and in our hands it abolishes the rapamycin-induced induction of LC3 II (Figure S1). Aliquots of the medium were taken at 14 h and proteins in the medium were precipitated with 20% trichloroacetic acid for 20 min on ice and centrifuged (10,000×g, 10 min, 4°C). Radioactivity in the supernatant (representing degraded proteins) and pellet (representing undegraded proteins) was measured in a liquid scintillation counter (Wallac T414, PerkinElmer Life Sciences). At the same time point, cells were also harvested and lysed with 0.1% NaOH. Proteolysis was expressed as the percentage of the initial total acid precipitable radioactivity (protein) in the cell lysates transformed to acid-soluble radioactivity (amino acids and small peptides) in the medium during the incubation. Baf or NH_4_Cl-inhibited degradation represented total lysosomal degradation, whereas 3MA-inhibited proteolysis represented macroautophagic degradation. Relative CMA-dependent degradation was calculated as total lysosomal minus macroautophagic-dependent degradation [14], as microautophagy is not thought to play a significant role in bulk lysosomal protein degradation. In addition CMA activity is routinely measured in this fashion in our laboratory and others [13], [14]; [29]. Such activity is drastically and commensurately altered with molecular manipulation of LAMP-2A expression [14; Xilouri et al., manuscript submitted], confirming that it reflects CMA-related activity. We cannot exclude the possibility that this measurement includes a small component of microautophagy.

### Western Immunoblotting

Cultured cells (differentiated neuroblastoma SH-SY5Y cells or differentiated WT ASYN ± dox cells or embryonic rat cortical neurons), were washed twice in cold PBS and then harvested in STET lysis buffer (50 mM Tris, pH 7,6, 150 mM NaCl, 1% Triton X-100, 2 mM EDTA) with protease inhibitors. Lysates were centrifuged at 10.000×g for 10 min at 4°C. Protein concentrations were determined using the Bradford method (Bio-Rad, Hercules, CA, USA). Twenty to thirty micrograms of lysates were mixed with 4x Laemmli buffer prior to running on 10% (for LAMP-2A, Hsc70), 12% (for ASYN) or 15% (for LC3) SDS–polyacrylamide gels. Following transfer to a nitrocellulose membrane, blots were probed with the following antibodies: polyclonal ASYN C-20 (Santa Cruz Biotechnology), polyclonal LC3 (Molecular Probes), polyclonal p62 (Molecular and Biological Laboratories), polyclonal rat LAMP-2A (Zymed, Invitrogen), polyclonal human LAMP-2A (Abcam), polyclonal Hsc70 (Abcam), polyclonal ERK (BD Biosciences, San Jose, CA, USA), and monoclonal β-actin (Santa Cruz Biotechnology). Blots were probed with horseradish peroxidase-conjugated secondary antibodies and visualized with enhanced chemiluminescence substrate (ECL) following exposure to Super RX film (FUJI FILM, Europe GmbH, Germany). After scanning the images with Adobe Photoshop 7,0, Gel analyzer software 1,0 (Biosure, Greece) was used to quantify the intensity of the bands.

### Statistical Analysis

Statistical analysis was performed using One-way ANOVA with a post hoc Tukey’s. Values of p<0,05 were considered significant. All statistical analyses were performed using the GRAPHPAD PRISM 4 suite of software (GraphPad Software, Inc., San Diego, CA, USA).

## Results

### ASYN Levels Show No Change after CBE Treatment in Differentiated SH-SY5Y Cells

**Figure 1:**
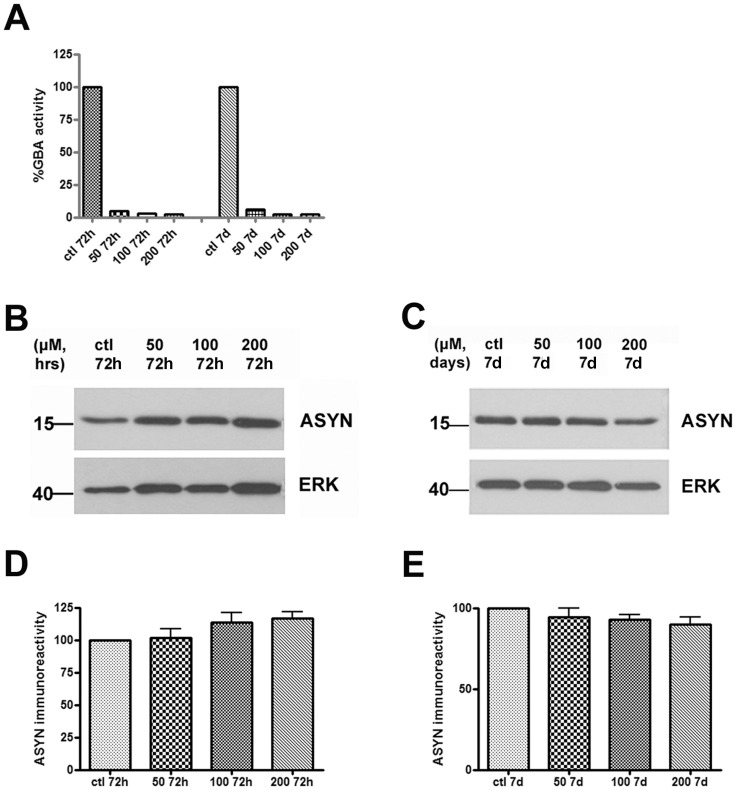
ASYN levels remain unchanged following GCase inhibition in differentiated SH-SY5Y cells. (A) Differentiated SH-SY5Y cells were exposed to increasing doses of CBE (50, 100, 200 µΜ) for 72 h and 7 days respectively. Untreated cells served as control (ctl). Cell pellets were assessed for GCase enzymatic activity at the aforementioned doses and time points. (B–C) Cell lysates were separated with SDS-PAGE and immunoblotted with the C-20 polyclonal antibody to ASYN. ERK = loading control. (D–E) Graphs show quantification of ASYN immunoreactivity (vs ERK) via densitometric analysis at 72 h and 7 day time point respectively. Statistical analysis made via One Way Anova showed no significant difference in ASYN levels at the given doses and time points (n = 8).

Previous data have shown that both pharmacological and molecular inhibition of GCase activity can lead to increased ASYN levels in *in vitro* systems [22], [23]. To analyze the effect of GCase inhibition upon ASYN levels we inhibited GCase activity by the use of the specific inhibitor CBE. We used a well-established cell line available in our laboratory, the neuroblastoma SH-SY5Y cells. These cells assume a neuron-like phenotype upon differentiation and express both ASYN and GCase. The first step was to assay GCase activity in these cells cultured in the presence of the CBE inhibitor for various periods of time (72 h, 7 days) and at various doses of the inhibitor (50, 100, 200 µΜ). We observed a dramatic inhibition of GCase enzymatic activity, even at the lower dose of 50 µM, following a 72 h exposure to CBE (∼ 95%) (Figure 1A). We then evaluated ASYN levels by Western immunoblot analysis under these conditions. No changes in ASYN levels were observed even at the higher dose and exposure time of CBE (200 µM, 7 days) (Figure 1B–E). Similar results were obtained in non-differentiated SH-SY5Y cells (data not shown). These data demonstrate that inhibition of the GCase is not sufficient to alter ASYN levels at the given time points in neuronal SH-SY5Y cells.

### Inhibition of GCase Activity does not Compromise Lysosomal Function in Differentiated SH-SY5Y Cells

As inhibition of GCase activity appeared to not affect ASYN levels in differentiated SH-SY5Y cells at the given doses and time points, we next examined whether GCase inhibition could affect lysosomal function.

**Figure 2:**
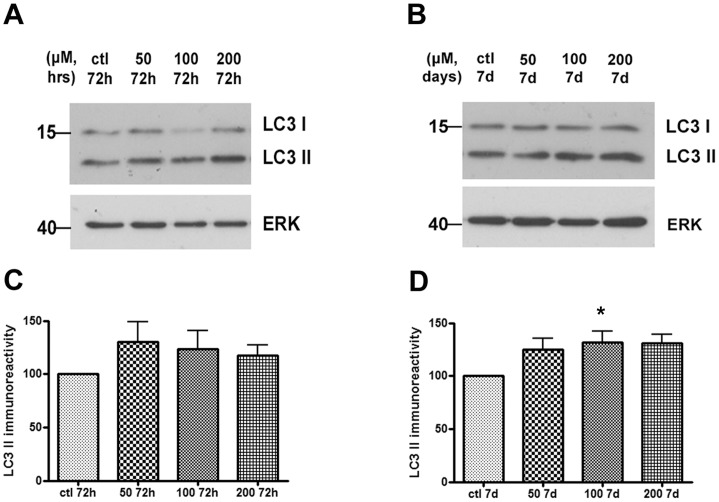
LC3 II levels slightly increase following GCase inhibition in differentiated SH-SY5Y cells. (A–B) Differentiated SH-SY5Y cells were exposed to increasing doses of CBE (50, 100, 200 µΜ) for 72 h and 7 days respectively. Untreated cells served as control (ctl). Cells were lysed, separated with SDS-PAGE and immunoblotted with the LC3 polyclonal antibody. ERK = loading control. (C–D) Graphs show quantification of LC3 II immunoreactivity (vs. ERK) via densitometric analysis at 72 h and 7 day time point respectively. Statistical analysis made via One Way Anova showed a significant increase in LC3 II levels for the 100 µΜ dose at the 7 day time point only, compared to ctl cultures (P<0,05; n = 8).

Lysosomal function has been found to be compromised upon partial depletion of the lysosomal form of GCase and in GD iPS neurons *in vitro* [23]. In a first level, we aimed to assess changes in LC3 II levels (an autophagosome marker per se) in differentiated SH-SY5Y cells. Increases in the conversion of LC3 I to LC3 II are a strong indication of accumulation of autophagosomes, which could occur either due to induction of macroautophagy or to inhibition of lysosomal function downstream of autophagosome formation [34]. To test this, we used the well-established general lysosomal inhibitor bafilomycin (Baf) which inhibits lysosomal acidification and prevents fusion of autophagosomes with lysosomes, thus leading to accumulation of autophagosomes and LC3 II (Figure S2). LC3 II levels were evaluated by Western blot analysis in differentiated SH-SY5Y cells upon treatment with CBE at different time points (72 h, 7 days) and doses (50, 100, 200 µΜ). We observed a trend for a slight increase of the LC3 II protein at all time-points and doses tested; however, this reached statistical significance only for the dose of 100 µM at 7 days (Figure 2). We also examined the levels of CMA markers, the lysosome-associated membrane protein type 2A (LAMP-2A) and the lysosomal Heat shock cognate protein of 70 kDa (Hsc70) [35]–[37] in the presence of the CBE inhibitor. We detected no alterations in the levels of the aforementioned proteins (Figure S3A).

**Figure 3:**
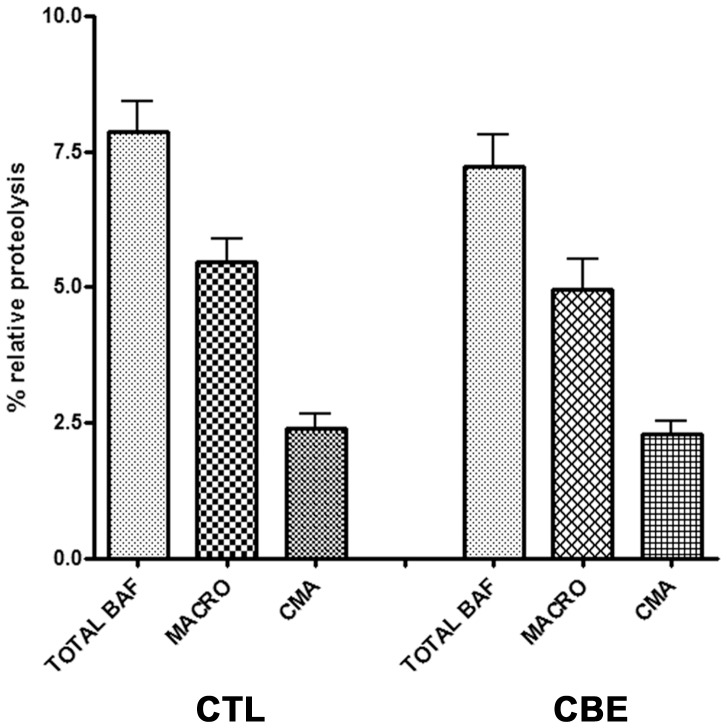
Lysosomal activity is not compromised following GCase inhibition in differentiated SH-SY5Y cells. Differentiated SH-SY5Y cells were treated or not (control cells) with CBE (200 µm) for 5 days. Following this, cells were radiolabelled with [^3^H] leucine supplemented with either 3MA (10 mM) or Baf (500 nM) and then assayed for long-lived protein degradation as described analytically in “Materials and Methods”. Untreated cells were also used as control (ctl). Values are the mean ±SE of two independent experiments that were represented in triplicate per condition. Statistical analysis made via One Way Anova revealed no difference in the activity of the different lysosomal pathways between CBE-treated and control cells.

Next, in order to examine lysosomal function per se, we measured the proteolysis of long-lived proteins in these cells with or without CBE treatment. This is a well-established method to monitor deviations in the lysosomal degradation capacity. Cells were radiolabelled with [^3^H] leucine in a pulse-chase experiment and then treated with the general lysosomal inhibitor bafilomycin (Baf) or the macroautophagy inhibitor 3 methyladenine (3MA) respectively. Untreated cells served as controls. These experiments revealed no alterations in the lysosomal degradation pathways, including macroautophagy (Figure 3). To further assess the possibility of subtle dysfunction of lysosomes, we examined the levels of p62, a sensitive indicator of macroautophagic failure [38]; levels of p62 were unaltered in these experiments with CBE application (Figure S4), further reinforcing the idea that profound GCase inhibition fails to lead to significant lysosomal/autophagic dysfunction.

### ASYN Levels Show No Change after CBE Treatment in Embryonic Rat Cortical Cultures

**Figure 4:**
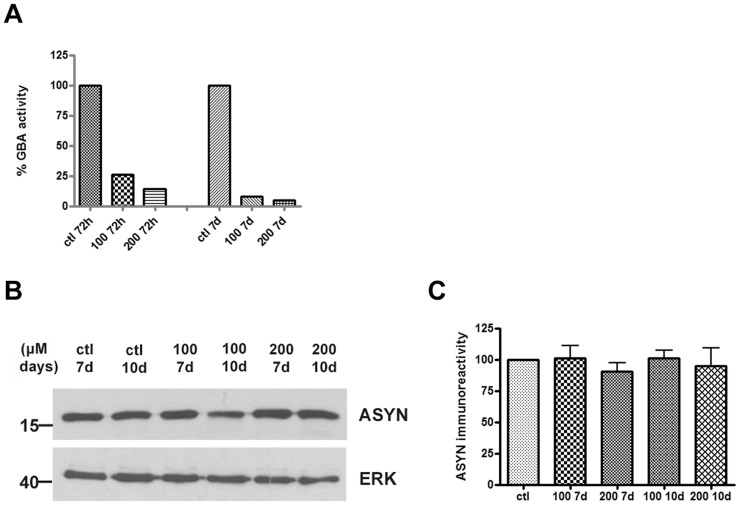
ASYN levels remain unchanged following GCase inhibition in rat embryonic cortical cultures. (A) Rat cortical neuron cultures were prepared from embryonic day 18 (E18) rat foetuses and were exposed at the 6^th^ day of culture to increasing doses of CBE (100, 200 µΜ) for 72 h and 7 days respectively. Untreated cells served as control (ctl). Cell pellets were assessed for GCase enzymatic activity at the aforementioned doses and time points. (B) Cell lysates were separated with SDS-PAGE and immunoblotted with the C-20 polyclonal antibody to ASYN. ERK = loading control. (C) Graph shows quantification of ASYN immunoreactivity (vs ERK) via densitometric analysis at the 7 and 10 day time points respectively. Statistical analysis made via One Way Anova showed no significant difference in ASYN levels at the given doses and time points (n = 6 samples/2 different culture preparations with 3 independent biological samples per condition).

Given the lack of change of ASYN levels with CBE treatment in differentiated SH-SY5Y cells, we wished to investigate whether we could detect any changes in primary neuron cell cultures. We used embryonic (E18) rat cortical cultures as they represent a homogenous source of near-pure post-mitotic neurons. Again, we measured GCase activity in these cultures upon treatment with CBE at different time points (72 h, 7 days) and doses (100, 200 μΜ). We observed strong inhibition of GCase activity in this cell culture system as well, even at the lower dose of 100 µM, at 72 h following exposure to CBE; this inhibition was more dramatic at the highest dose of 200 µM at 7 days (95%) (Figure 4A). ASYN levels showed no change upon treatment with the CBE inhibitor (100, 200 µΜ) at 7 and 10 days respectively (Figure 4B–C), similarly to differentiated SH-SY5Y cells. These results suggest that GCase inhibition does not affect ASYN levels, at least in cultured neuronal cells, and over the relatively short time frames utilized in this study.

### Macroautophagy and CMA Markers Show No Alteration after CBE Treatment in Embryonic Rat Cortical Cultures

**Figure 5:**
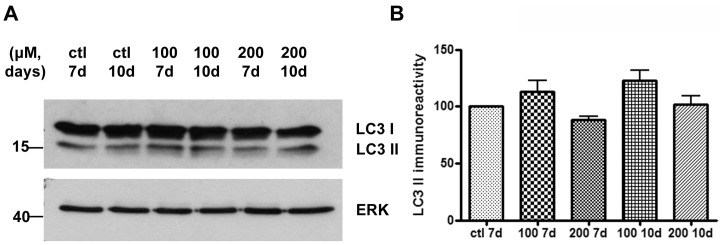
LC3 II shows no change following GCase inhibition in rat embryonic cortical cultures. (A) Cultured cortical neurons (E18) were exposed to increasing doses of CBE (100, 200 µΜ) for 7 and 10 days respectively. Untreated cells served as control (ctl). Cells were lysed, separated with SDS-PAGE and immunoblotted with the LC3 polyclonal antibody. ERK = loading control. (B) Graphs show quantification of LC3 II immunoreactivity (vs. ERK) via densitometric analysis at the 7 and 10 day time points respectively. Statistical analysis made via One Way Anova showed no difference in the LC3 II levels for the corresponding doses and time points of CBE in comparison to the control samples (n = 6 samples/2 different culture preparations with 3 independent biological samples per condition).

Following the same strategy as for SH-SY5Y neuroblastoma cells, we next assessed the effect of GCase inhibition on LC3 II levels in embryonic rat cortical cultures. The ability to detect accumulation of autophagosomes in this primary neuron culture system was verified by the use of the general lysosomal inhibitor ammonium chloride (NH_4_Cl) (Figure S2). LC3 II levels were then analyzed by Western blot upon application of the CBE inhibitor (100, 200 µΜ) at 7 and 10 days respectively. No alteration in LC3 II levels was observed in any of the given time points (Figure 5). CMA markers were also assessed revealing once again no alterations (Figure S3B). Given these results and the fact that no significant lysosomal impairment was evident in the differentiated SH-SY5Y cells upon CBE exposure, we did not further assess proteolysis of long-lived proteins in these cultures.

### GCase Inhibition does not Affect The Formation or Clearance of ASYN Oligomers

**Figure 6:**
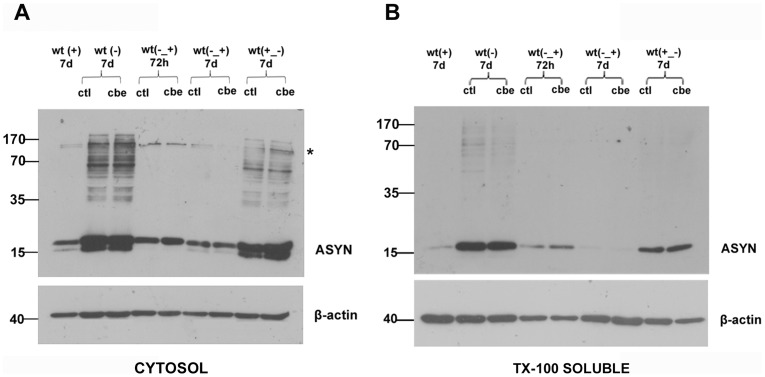
GCase inhibition does not affect ASYN-specific HMW species in differentiated WT ASYN cells. A stable inducible Tet-Off SH-SY5Y neuroblastoma cell line overexpressing WT ASYN was used in this assay. Initially cells were cultured in the presence (+) or absence (−) of dox (2 µg/mL) for 7 days. Subsequently cells were differentiated for 5 days and then were exposed to CBE (200 µM) at different conditions. Untreated cells were also used (ctl). WT (+) 7 d: differentiated WT ASYN cells expressing basal levels of ASYN in the presence of dox for 7 days, WT (−) 7 d: differentiated WT ASYN overexpressing cells in the absence of dox, treated with CBE, or not (ctl) for 7 days. WT (−_+) 72 h: differentiated WT ASYN overexpressing cells were switched to +dox conditions for 72 h along with the presence or not of CBE, WT (−_+) 7 d: differentiated WT ASYN overexpressing cells were switched to +dox conditions for 7 days, along with the presence or not of CBE. WT (+_−) 7 d: differentiated WT ASYN cells expressing basal levels of ASYN were switched to -dox conditions for 7 days, along with the presence or not of CBE. Cell lysates were separated with SDS-PAGE and immunoblotted with the C-20 polyclonal antibody to ASYN. β-actin = loading control. At no condition was there any difference in the presence or relative amount of ASYN monomers or ASYN-specific High Molecular Weight (HMW) species between the CBE and control-treated cells in the cytosol (A) and the membrane-associated, Triton X-100 soluble fraction (B) respectively. A doublet that is also present in the+dox conditions represents no specific immunolabeling (designated with an asterisk).

As GCase inhibition did not appear to increase the monomeric levels of ASYN, we next assessed the effects of such inhibition on ASYN oligomers. Others have presented data suggesting that GCase deficiency can influence the aggregation of ASYN *in vitro* by stabilizing oligomeric intermediates [23]. For this, we used a doxycycline inducible Tet- Off system for ASYN. In the absence of doxycycline ASYN is overexpressed and creates oligomeric species (Off system), whereas in the presence of doxycycline ASYN is expressed at basal levels. ASYN monomeric and oligomeric levels were assessed in the presence or absence of CBE in this inducible system via Western blot analysis at different stages of the formation or clearance of these species. We were not able to detect alterations in ASYN oligomeric or monomeric species upon treatment with CBE (Figure 6). Of note, ASYN was not detectable in the SDS or urea-soluble fraction in these cells (data not shown) and this is consistent with the fact that we do not detect aggregated ASYN by immunostaining in these cultures. These results support the notion that profound GCase inhibition is not sufficient to influence ASYN in neuronal cell cultures, at least within the time frame employed in the current experiments.

## Discussion

A variety of genetic, pathological and clinical studies have suggested a potential biological link between Type 1 Gaucher (GD) and Parkinson’s disease (PD). Interestingly, this link has been proposed to be attributed, at least to an extent, to the interplay between GCase and ASYN [17]–[20], [23]. Gain-of-function, loss-of-function and prion theories have been proposed to date, describing either a direct or an indirect interaction between GCase and ASYN, but all with significant limitations [18]–[23], [39]–[43]. In the present study, we demonstrate that profound deficiency of GCase activity via pharmacological inhibition was not sufficient, within the time frame examined, to alter ASYN levels, neither in neuronally differentiated SH-SY5Y cells, nor in primary cortical neuronal cultures. In support of the above results, fractionation studies in a doxycycline inducible Tet-Off system for ASYN revealed again no changes in the clearance or formation of oligomeric forms of ASYN following GCase inhibition. Further, GCase inhibition did not appear to significantly compromise lysosomal function in the above cell culture models. We observed only a slight increase of the LC3 II protein levels at one specific time-point and no distortion in any of the lysosomal degradation pathways, including macroautophagy.

Taken together, our results demonstrate that GCase deficiency is not sufficient to alter ASYN levels (monomeric or oligomeric) in differentiated SH-SY5Y cells and rat cortical neuronal cultures. Our findings thus contrast with a study showing that loss of GCase activity through a similar pharmacological approach led to enhanced ASYN levels in neuroblastoma cells [22]. Mazzulli et al. [23] showed that significant, but partial, reduction of the levels of the GCase protein was sufficient to induce protein levels of ASYN, but this study is not directly comparable to ours, as in our experiments GCase protein is still present, but dysfunctional due to the pharmacological inhibition. There are certainly limitations inherent to the approach we have used, and in particular the use of cell systems (cell lines or primary cultures) and the short time frames of monitoring for an effect, which could potentially mask the outcome of an interaction between loss-of-function of GCase and ASYN, which otherwise might have been obvious in longer-term *in vivo* settings. Our findings are more in accord with those of Cullen et al. [21], who did not find alterations in ASYN levels following profound chemical inhibition of GCase activity by CBE in cell lines. In the current work, we have extended these findings by assessing lysosomal function in the face of GCase inhibition. Our results indicate no significant impairment of the lysosomal degradation machinery. Mazzulli et al. [23] in contrast, found that loss of GCase protein levels and resultant loss of activity was associated with lysosomal dysfunction, and posited that such lysosomal dysfunction led to secondary ASYN accumulation. Methodological differences, in particular in the cell types tested, may account for the observed discrepancies. Alternatively, loss of GCase protein levels may have other effects on lysosomal function, independent of the known GCase enzymatic activity.

Our results suggest that the scenario of a direct sequence of events of GCase inhibition leading to lysosomal dysfunction, and this in turn leading to ASYN accumulation, is unlikely. It could be instead that the link of GBA mutations to ASYN accumulation in PD involves gain-of-function mechanisms, rather than loss-of-function, whereby GBA mutations could potentially result in an unstable or misfolded protein, which in turn could contribute to the enhanced accumulation and aggregation of ASYN. In addition, subsequent substrate accumulation could synergistically add to the pathology of ASYN. This would be in accord with the data of Cullen et al. [21], who found enhanced levels of ASYN in situations where mutant GBA was overexpressed, without loss of the enzymatic activity. Alternatively, more prolonged GCase inhibition may be needed to lead to ASYN accumulation and/or aggregation. This could occur secondary to the accumulation of GCase substrates, as suggested by our finding that ASYN has an increased tendency to oligomerize in erythrocyte membranes of GD patients, compared to controls [44]; it has been demonstrated that glucosylceramide (GluCer), a GCase substrate, accumulates in GD erythrocyte membranes [45], and we have suggested that such accumulation may underlie the increased oligomerization of ASYN [44]. Another explanation could be that the interaction between aberrant GCase and ASYN is in fact indirect and that contribution of other gene modifiers is pivotal for the development of the disease. This would account for the discrepancies observed across studies, and also explain why only a fraction of carriers and GD patients develop PD.

Concluding, to date, the contribution of an association between GCase deficiency and ASYN to PD and related disorders remains elusive. Our study questions a simplistic model in which GCase inhibition leads to ASYN accumulation/aggregation via lysosomal dysfunction, as neither of these phenomena occur in neuronal cells exposed to profound GCase inhibition. A future challenge will be to unmask other risk factors that, together with aberrant GCase, synergistically add to the pathology of the disease, thus providing new insights for therapeutic intervention.

## Supporting Information

Figure S1 **Complete inhibition of the rapamycin-induced induction of LC3 II with 3MA in cortical neuron cultures.** Rat embryonic cortical cultures (E18) on their 7^th^ day of culture were exposed either to the macroautophagy inhibitor 3MA (10 mM) or the mTOR inhibitor rapamycin (activator of macroautophagy) (500 nM) (rap) or in 3MA together with rapamycin (rap+3MA). Untreated cells served as control (ctl). 24 h later the cells were harvested for SDS-PAGE analysis against the polyclonal LC3 antibody. ERK = loading control. (A) Increased levels of the LC3 II protein were found in the presence of rapamycin, whereas in the presence of the rapamycin together with 3MA the LC3 II levels decreased, reaching the levels of the 3MA-alone treated cells. (B) Statistical analysis made via One Way Anova showed significant difference between 3MA vs ctl (*P<0,05), 3MA vs rap (^##^p<0,001) and rap+3MA vs rap (*P<0,01).
(TIF)

Figure S2 **Induction of autophagic vacuoles in differentiated SH-SY5Y cells and cortical neuron cultures.** Differentiated SH-SY5Y cells or rat embryonic cortical cultures (E18) on their 6^th^ day of culture were exposed to the general lysosomal inhibitor bafilomycin (Baf) (500 nM) or ammonium chloride (NH_4_Cl) (20 mM) respectively overnight. Untreated cells served as control (ctl). Cells were lysed, separated with SDS-PAGE and immunoblotted with an LC3 polyclonal antibody. ERK = loading control. Increase in the conversion of the LC3 I to LC3 II was evident in both differentiated SH-SY5Y cells and cultured cortical neuron cultures.
(TIF)

Figure S3 **No alteration in LAMP-2A and Hsc70 levels in differentiated SH-SY5Y cells and cortical neuron cultures.** Differentiated SH-SY5Y cells or rat cultured cortical neurons (E18) on their 6^th^ day of culture, were exposed to increasing doses of CBE (50, 100, 200 µΜ) for 7 days. Untreated cells served as control (ctl). Cells were lysed, separated with SDS-PAGE and immunoblotted with a LAMP-2A (human or rat) and Hsc70 polyclonal antibody. ERK = loading control. No significant alterations in the LAMP-2A and Hsc70 levels were evident either in the differentiated SH-SY5Y cells (A) or primary cortical neurons (B). The band corresponding to LAMP-2A protein is indicated by an arrow. Similar results were achieved in one or two other independent experiments in the differentiated SH-SY5Y cells and primary cortical cultures respectively.
(TIF)

Figure S4 **No alteration in p62 levels in differentiated SH-SY5Y cells.** Differentiated SH-SY5Y cells were exposed to increasing doses of CBE (50, 100, 200 µΜ) for 72 h and 7 days respectively. Untreated cells served as control (ctl). Cells were lysed, separated with SDS-PAGE and immunoblotted with a p62 polyclonal antibody ERK = loading control. No significant differences in p62 levels were detected at the given doses and time points (representative of 4 experiments).
(TIF)
