# Correction: Loss of β-Glucocerebrosidase Activity Does Not Affect Alpha-Synuclein Levels or Lysosomal Function in Neuronal Cells

**DOI:** 10.1371/journal.pone.0252975

**Published:** 2021-06-04

**Authors:** Georgia Dermentzaki, Evangelia Dimitriou, Maria Xilouri, Helen Michelakakis, Leonidas Stefanis

Following the publication of this article [1] concerns were raised regarding the β-actin blot presented in Fig 6A. Horizontal discontinuities and repetitive elements were detected in the background directly below the bands representing β-actin signal. The original β-actin blot presented additional bands underneath the β-actin results visible in lanes 1–3, and 6–9, which were removed from the panel to improve the clarity of the presented blot, and only the top bands were used for quantification. The removing of the bands beneath the β-actin results contravenes *PLOS ONE*’s figure preparation guidelines, instead the lower bands should have been included in the published panel, and the text should have commented on the inconsistency.

**Fig 6:**
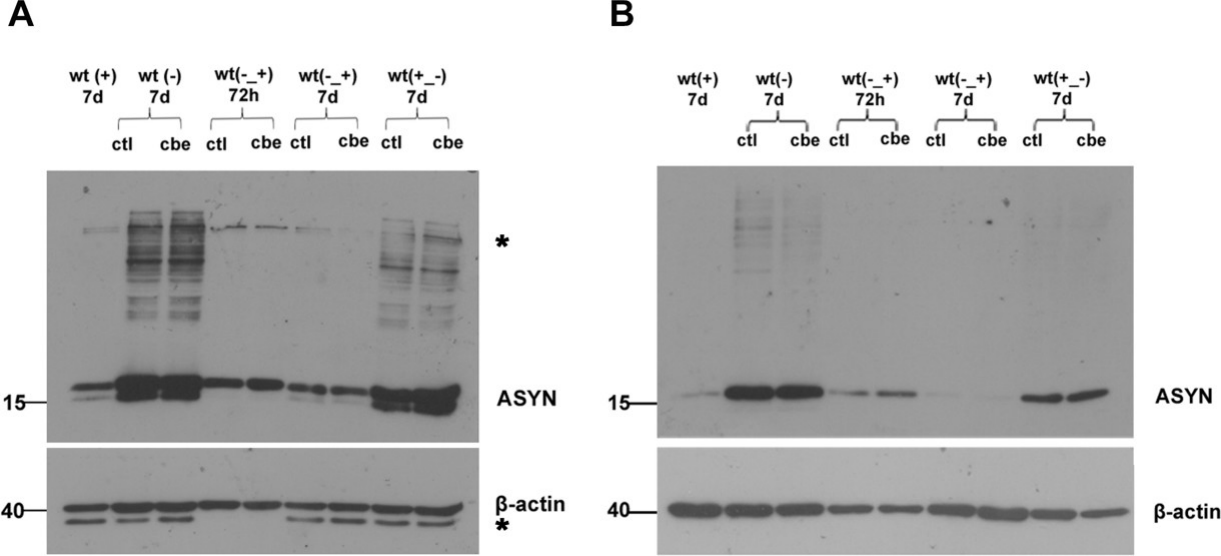
GCase inhibition does not affect ASYN-specific HMW species in differentiated WT ASYN cells. A stable inducible Tet-Off SH-SY5Y neuroblastoma cell line overexpressing WT ASYN was used in this assay. Initially cells were cultured in the presence (+) or absence (−) of dox (2 μg/mL) for 7 days. Subsequently cells were differentiated for 5 days and then were exposed to CBE (200 μM) at different conditions. Untreated cells were also used (ctl). WT (+) 7 d: differentiated WT ASYN cells expressing basal levels of ASYN in the presence of dox for 7 days, WT (−) 7 d: differentiated WT ASYN overexpressing cells in the absence of dox, treated with CBE, or not (ctl) for 7 days. WT (−_+) 72 h: differentiated WT ASYN overexpressing cells were switched to +dox conditions for 72 h along with the presence or not of CBE, WT (−_+) 7 d: differentiated WT ASYN overexpressing cells were switched to +dox conditions for 7 days, along with the presence or not of CBE. WT (+_−) 7 d: differentiated WT ASYN cells expressing basal levels of ASYN were switched to -dox conditions for 7 days, along with the presence or not of CBE. Cell lysates were separated with SDS-PAGE and immunoblotted with the C-20 polyclonal antibody to ASYN. β-actin  =  loading control. At no condition was there any difference in the presence or relative amount of ASYN monomers or ASYN-specific High Molecular Weight (HMW) species between the CBE and control-treated cells in the cytosol (A) and the membrane-associated, Triton X-100 soluble fraction (B) respectively. A doublet that is also present in the+dox conditions for ASYN represents non-specific immunolabeling (designated with an asterisk); An extra band below β-actin most probably represents a post-translational modification or other actin isoforms (designated with an asterisk).

This Correction notice is issued to update the Fig 6 results to ensure the experimental data are presented in accordance with the journal’s figure guidelines. The original blots underlying the panels presented in Fig 6 are provided in the Supporting Information files S1–S4 Files. The raw data underlying all other results reported in the article are available upon request.

## Supporting information

S1 File Original blot underlying Fig 6 Cytosol ASYN. (BMP)

S2 File Original blot underlying Fig 6 Cytosol β-actin. (TIF)

S3 File Original blot underlying Fig 6 TX-100 Soluble ASYN. (BMP)

S4 File Original blot underlying Fig 6 TX-100 Soluble β-actin. (TIF)
