# Supplementary figures and images for: Loss of β-Glucocerebrosidase Activity Does Not Affect Alpha-Synuclein Levels or Lysosomal Function in Neuronal Cells
Source: PLoS One. 2013 Apr 8;8(4):e60674. doi: 10.1371/journal.pone.0060674 (PMC3620326; doi:10.1371/journal.pone.0060674)

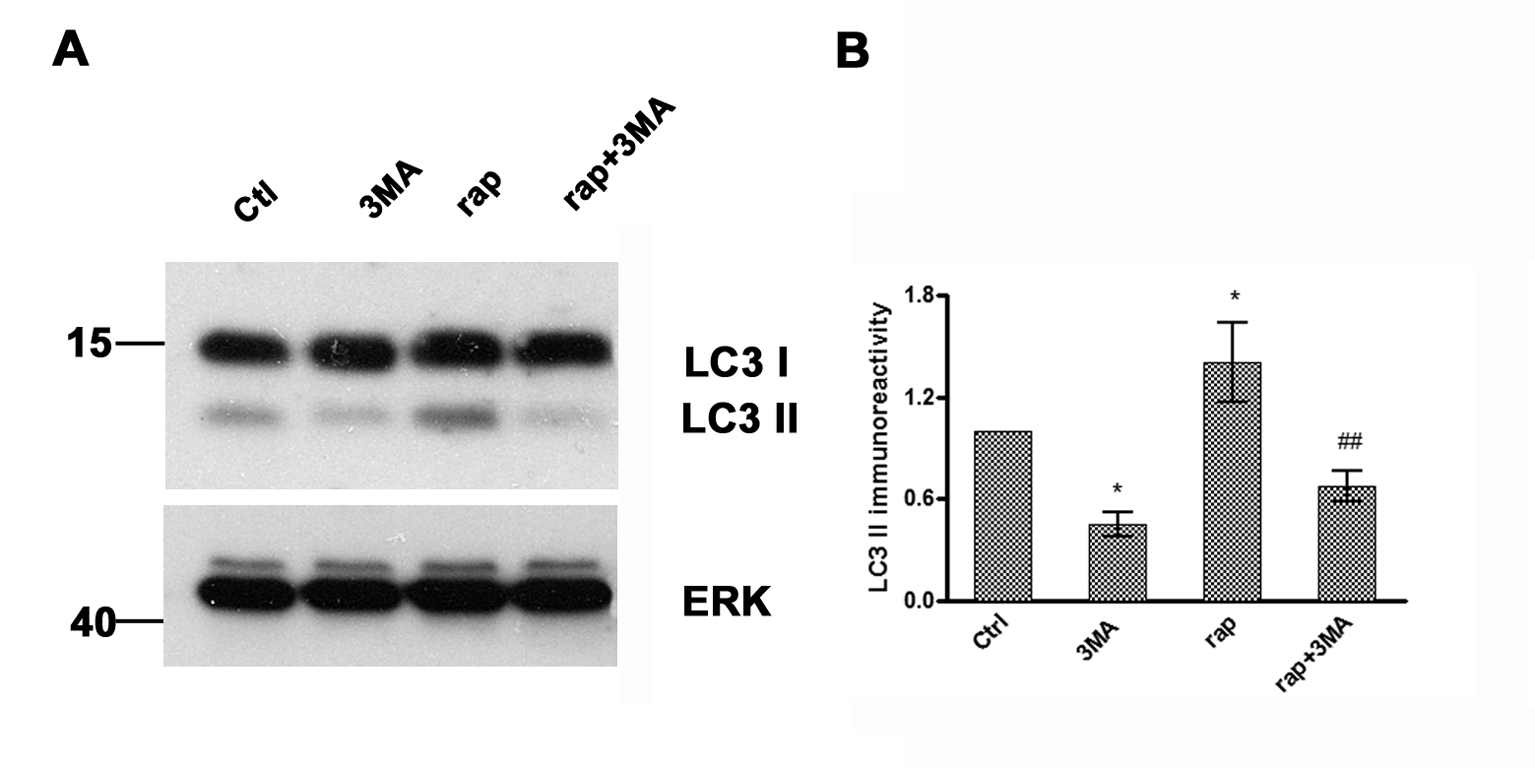

Supplement: Figure S1 — Complete inhibition of the rapamycin-induced induction of LC3 II with 3MA in cortical neuron cultures. Rat embryonic cortical cultures (E18) on their 7th day of culture were exposed either to the macroautophagy inhibitor 3MA (10 mM) or the mTOR inhibitor rapamycin (activator of macroautophagy) (500 nM) (rap) or in 3MA together with rapamycin (rap+3MA). Untreated cells served as control (ctl). 24 h later the cells were harvested for SDS-PAGE analysis against the polyclonal LC3 antibody. ERK = loading control. (A) Increased levels of the LC3 II protein were found in the presence of rapamycin, whereas in the presence of the rapamycin together with 3MA the LC3 II levels decreased, reaching the levels of the 3MA-alone treated cells. (B) Statistical analysis made via One Way Anova showed significant difference between 3MA vs ctl (*P<0,05), 3MA vs rap (##p<0,001) and rap+3MA vs rap (*P<0,01). (TIF) [file pone.0060674.s001.tif]

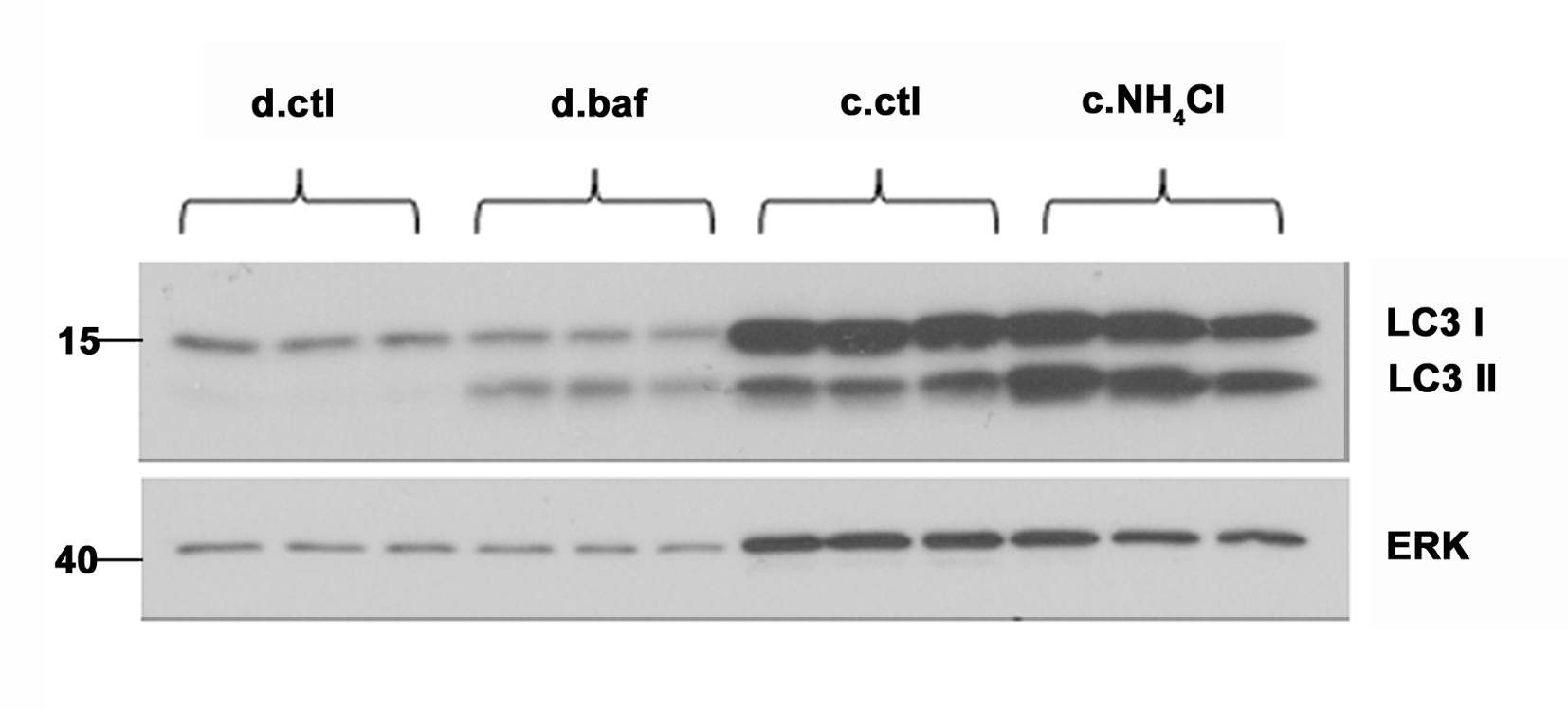

Supplement: Figure S2 — Induction of autophagic vacuoles in differentiated SH-SY5Y cells and cortical neuron cultures. Differentiated SH-SY5Y cells or rat embryonic cortical cultures (E18) on their 6th day of culture were exposed to the general lysosomal inhibitor bafilomycin (Baf) (500 nM) or ammonium chloride (NH4Cl) (20 mM) respectively overnight. Untreated cells served as control (ctl). Cells were lysed, separated with SDS-PAGE and immunoblotted with an LC3 polyclonal antibody. ERK = loading control. Increase in the conversion of the LC3 I to LC3 II was evident in both differentiated SH-SY5Y cells and cultured cortical neuron cultures. (TIF) [file pone.0060674.s002.tif]

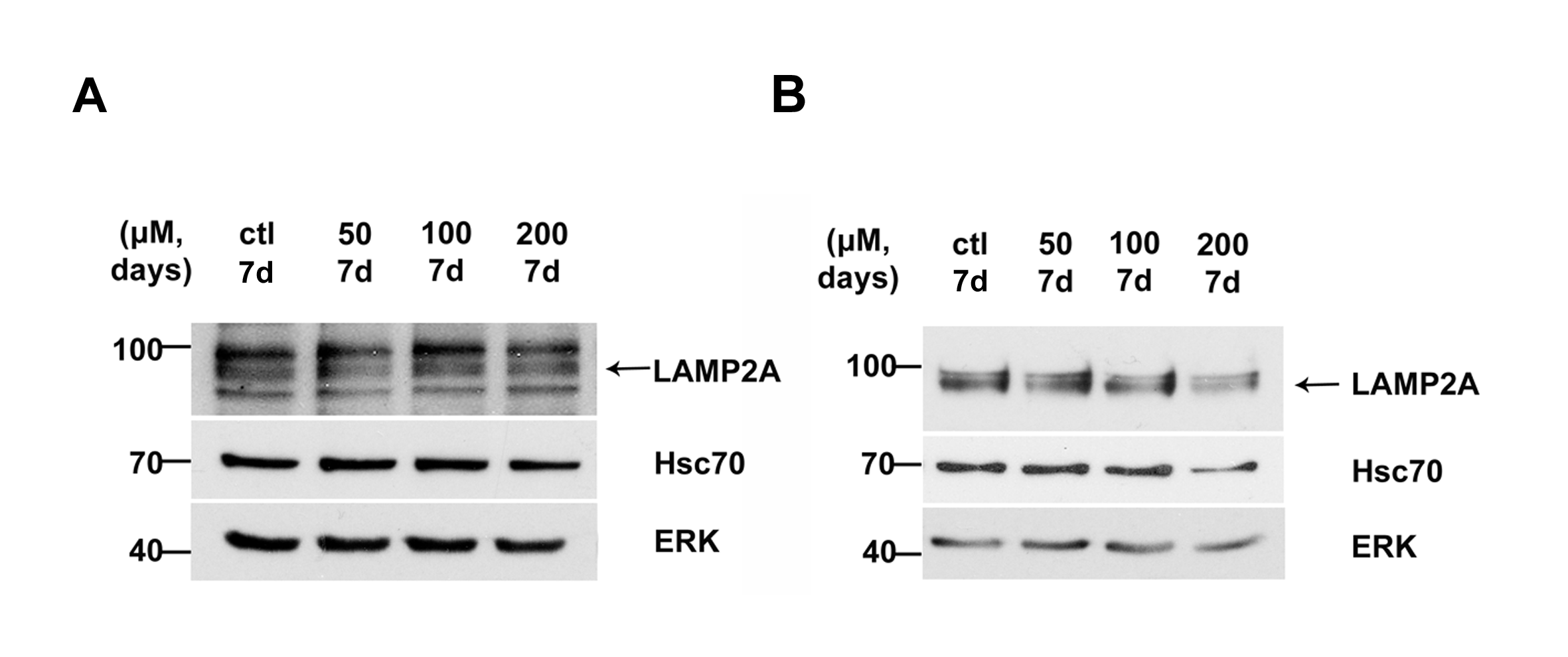

Supplement: Figure S3 — No alteration in LAMP-2A and Hsc70 levels in differentiated SH-SY5Y cells and cortical neuron cultures. Differentiated SH-SY5Y cells or rat cultured cortical neurons (E18) on their 6th day of culture, were exposed to increasing doses of CBE (50, 100, 200 µΜ) for 7 days. Untreated cells served as control (ctl). Cells were lysed, separated with SDS-PAGE and immunoblotted with a LAMP-2A (human or rat) and Hsc70 polyclonal antibody. ERK = loading control. No significant alterations in the LAMP-2A and Hsc70 levels were evident either in the differentiated SH-SY5Y cells (A) or primary cortical neurons (B). The band corresponding to LAMP-2A protein is indicated by an arrow. Similar results were achieved in one or two other independent experiments in the differentiated SH-SY5Y cells and primary cortical cultures respectively. (TIF) [file pone.0060674.s003.tif]

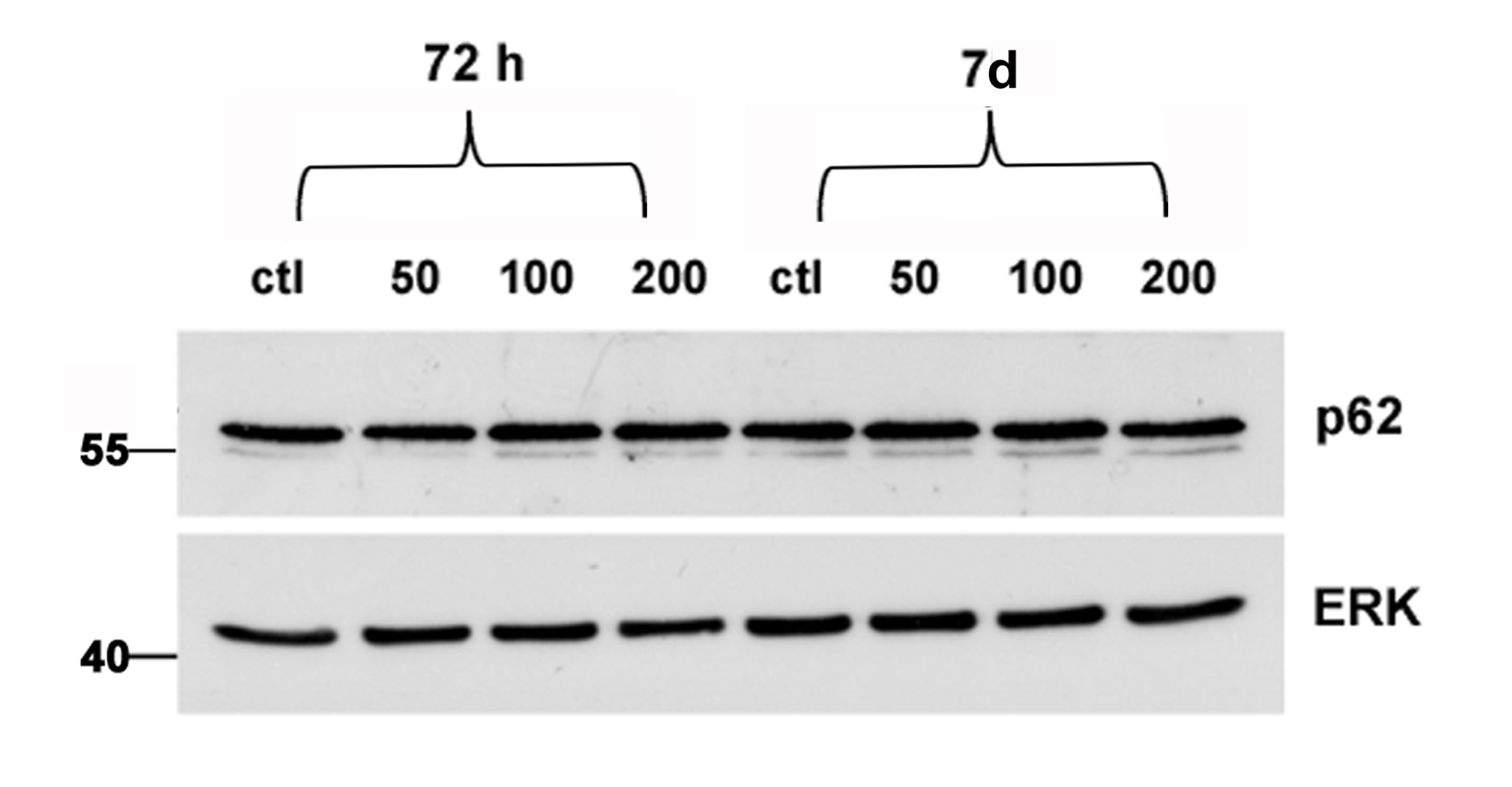

Supplement: Figure S4 — No alteration in p62 levels in differentiated SH-SY5Y cells. Differentiated SH-SY5Y cells were exposed to increasing doses of CBE (50, 100, 200 µΜ) for 72 h and 7 days respectively. Untreated cells served as control (ctl). Cells were lysed, separated with SDS-PAGE and immunoblotted with a p62 polyclonal antibody ERK = loading control. No significant differences in p62 levels were detected at the given doses and time points (representative of 4 experiments). (TIF) [file pone.0060674.s004.tif]
