# Supplementary figures and images for: Correction: Loss of β-Glucocerebrosidase Activity Does Not Affect Alpha-Synuclein Levels or Lysosomal Function in Neuronal Cells
Source: PLoS One. 2021 Jun 4;16(6):e0252975. doi: 10.1371/journal.pone.0252975 (PMC8177519; doi:10.1371/journal.pone.0252975)

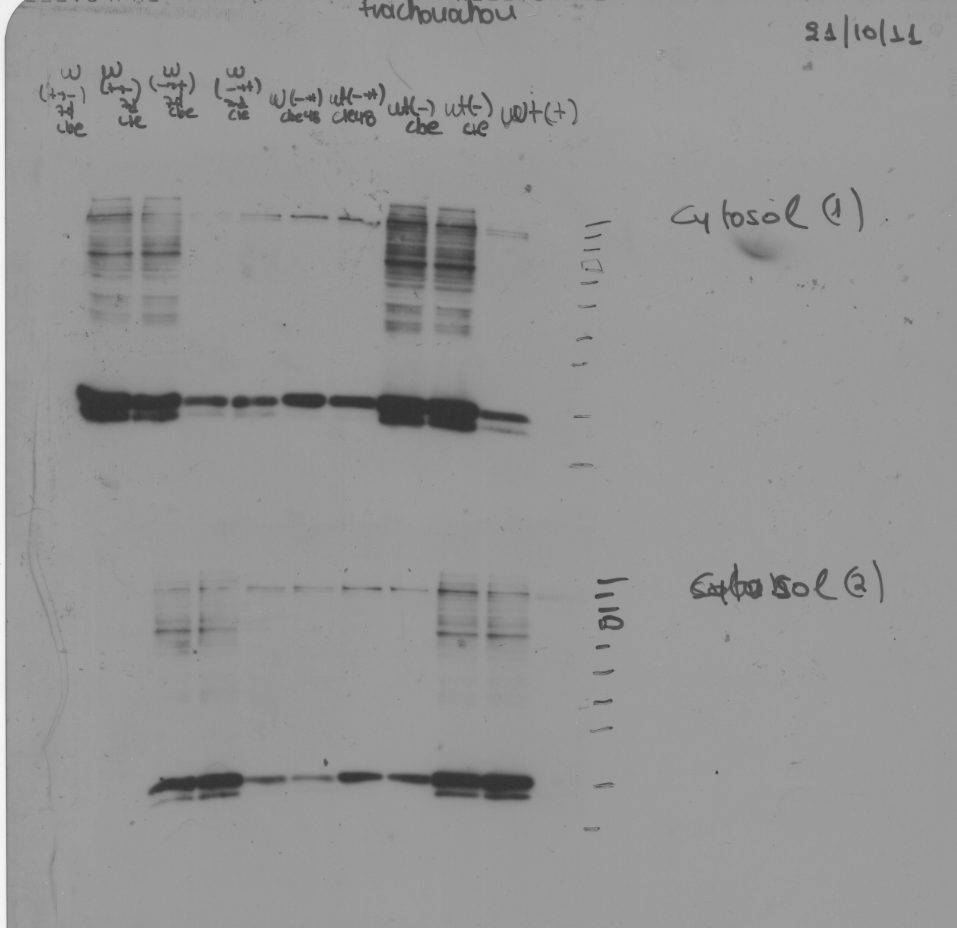

Supplement: S1 File — (BMP) [file pone.0252975.s001.bmp]

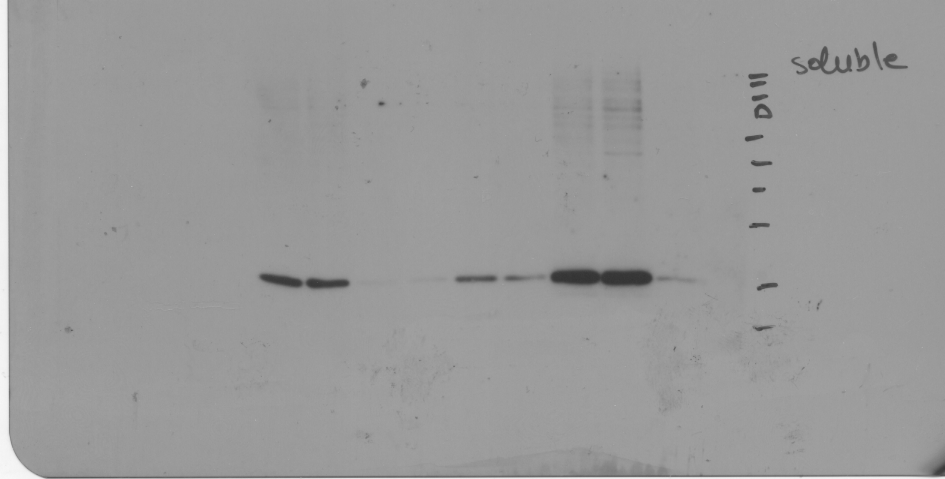

Supplement: S3 File — (BMP) [file pone.0252975.s003.bmp]
